# Arterial health during early childhood following abnormal fetal growth

**DOI:** 10.1186/s12887-021-02951-2

**Published:** 2022-01-14

**Authors:** Rasmus F.W. Olander, Johnny K.M. Sundholm, Sanna Suonsyrjä, Taisto Sarkola

**Affiliations:** 1 Children’s Hospital, Pediatric Research Center, University of Helsinki, and Helsinki University Hospital, Stenbäckinkatu 9, POB 347, FIN-00029 Helsinki, Finland; 2 Minerva Foundation Institute for Medical Research, Helsinki, Finland

**Keywords:** Adventitia, Arteries, Blood pressure, Carotid intima-media thickness, Child health, Fetal growth restriction, Ultrasonography, Arterial stiffness

## Abstract

**Background:**

Abnormal fetal growth is associated with increased cardiovascular risk in adulthood. We investigated the effect of fetal programming on arterial health and morphology during early childhood.

**Methods:**

We examined 90 children (median age 5.81 years, interquartile range: 5.67; 5.95), born small for gestational age with fetal growth restriction, large or appropriate for gestational age (SGA, N = 23, LGA, N = 19, AGA N = 48). We measured body composition, anthropometrics, blood pressure, pulse wave velocity (PWV), lipids, glucose and inflammatory markers, and assessed carotid, brachial, radial and femoral arterial morphology and stiffness using very-high resolution ultrasound (46–71 MHz).

**Results:**

LGA showed increased anthropometry, lean body mass and body mass index. SGA displayed decreased anthropometry and lean body mass. Blood pressure, PWV, carotid artery stiffness and blood work did not differ groupwise. Differences in lumen diameters, intima-media thicknesses (IMT) and adventitia thicknesses disappeared when adjusted for lean body mass and sex. In multiple regression models arterial dimensions were mainly predicted by lean body mass, with birth weight remaining associated only with carotid and brachial lumen dimensions, and not with IMTs. Carotid-femoral PWV was predicted by height and blood pressure only. No independent effect of adiposity was observed.

**Conclusions:**

Arterial dimensions in childhood associate with current anthropometrics, especially lean body mass, and sex, explaining differences in arterial layer thickness. We found no signs of fetal programming of cardiovascular risk or arterial health in early childhood.

**Supplementary Information:**

The online version contains supplementary material available at 10.1186/s12887-021-02951-2.

## Background

The gestational milieu, affecting fetal growth and development, has an impact on health in adulthood [1]. Low birth weight or being born small for gestational age (SGA), is associated with an increased cardiovascular risk profile and disease [2, 3], in adulthood. Conversely, the association between high birth weight, or being born large for gestational age (LGA), and cardiovascular risk is not as consistent [2–4].

The lifelong development of atherosclerosis begins in childhood and leads to cardiovascular disease, including peripheral artery disease, in adult age [5]. An adverse cardiovascular risk profile and morbidity has been linked to increased common carotid intima-media thickness (IMT) in teenagers and adults, whereas the relationship is not as clear during childhood [6, 7]. Carotid IMT, however, remains a significant source of information on cardiovascular risk when measured in a standardized manner [8], and being born SGA has been determined to be related with increased carotid IMT in early childhood [9].

Carotid IMT and other arterial dimensions have, to our knowledge, so far only been assessed in this setting using conventional ultrasound (< 15 MHz). However, conventional ultrasound has previously been shown to be inadequate for measuring these dimensions in infants and young children and instead very-high resolution ultrasound has been validated (VHRU; peak frequencies 46–71 MHz) as an accurate non-invasive method to assess minute superficial arterial layer thickness in this age group [10, 11]. It is thus still unclear whether the reported increased carotid IMT in children with altered fetal growth reflects an increase in cardiovascular risk profile, due to the use of conventional ultrasound in previous studies. Furthermore, when examined using VHRU the IMT of SGA and LGA infants has been demonstrated to mainly associate with body anthropometrics [12]. Arterial dimensions in early childhood, assessed using VHRU, are related to anthropometrics [13], with lean body mass (LBM) being a significant predictor [14]. We are the first to report on arterial dimensions during early childhood in the setting of abnormal fetal growth using methods validated for this age group.

We aimed to assess the impact of fetal programming, as seen in pre- and postnatal growth, on arterial health and cardiovascular risk during early childhood, by studying arterial structure, stiffness, blood pressure (BP), adiposity, blood glucose, lipids, and inflammatory markers. In addition, we investigated predictors for arterial dimensions and function in this setting. We hypothesized that current body size in general, and LBM in particular, would be the main predictor of arterial dimensions.

## Methods

### Study design, sample and setting

**Fig. 1:**
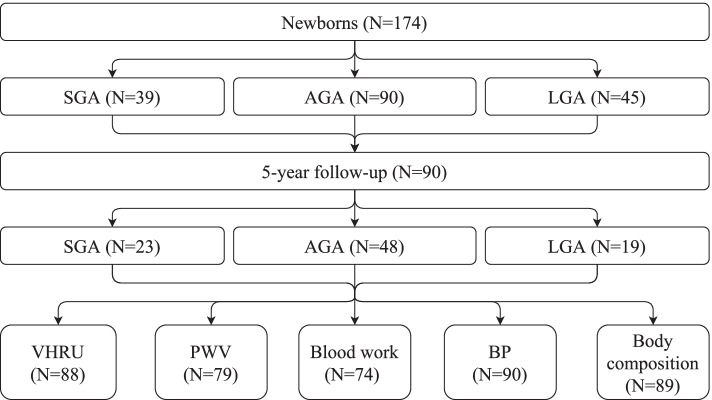
Groupwise distribution of patients and the number participating in each part of the study. SGA indicates small for gestational age, AGA appropriate for gestational age, LGA large for gestational age, VHRU very-high resolution ultrasound, PWV pulse wave velocity, and BP blood pressure

We recruited 174 newborns between November 2011 and January 2014 at the Women’s Hospital, Helsinki Finland to this longitudinal observational cohort study. The newborns, born at weeks 31–42, were recruited into three groups: SGA, LGA or appropriate for gestational age (AGA); defined as weight Z-score <-2 (SGA) and > + 2 (LGA), according to growth charts in use at that time [15]. As the birth weight of the SGA groups was below the 3rd percentile at recruitment, the group corresponded in its entirety to the criteria for fetal growth restriction (FGR), as set by the International Society of Ultrasound in Obstetrics and Gynecology practice guidelines [16]. During the follow-up period new fetal and childhood growth curves were published, and we used these to generate new Z-scores for birth weight and to track growth from primary health care visits [17–19]. The new Z-scores corresponded well to the group assignment done at birth, despite minor discrepancies for certain individuals. In order to maintain combability to the neonate stage, no reassignment was done between the groups. Patients with malformations, genetic or chromosomal abnormalities were excluded from the initial recruitment, as reported earlier [12, 20]. In this manuscript we report on the follow-up of 90 preschool children, examined between October 2017 and June 2019, outlined in Fig. 1. During the follow-up period, one child was reported as having been diagnosed with corpus callosum agenesia and one with mild asymmetric cerebral palsy affecting the upper body, both occurring in isolation. As the cardiovascular impact of these findings were minor, none of the subjects who agreed to the follow-up were excluded. Written informed consent was obtained from the children’s guardians. The Helsinki University Hospital Ethics Committee for gynecology and obstetrics, pediatrics and psychiatry approved the research protocol (138/13/03/03/2011 and HUS/2274/2016).

### Arterial morphology and stiffness

One skilled investigator obtained VHRU images (peak frequencies 46–71 MHz) using the Vevo MD system, and another blinded investigator analyzed these offline, using the VevoLAB 3.1.1. software (VisualSonics, Toronto, Canada). We examined the common carotid and femoral arteries bilaterally and the right brachial and radial arteries unilaterally. The carotid artery was examined 1 cm proximally of the carotid bulb, the radial artery 1 cm proximally of the palm, the brachial artery 5 cm proximally of the cubital fossa, and the femoral artery 2 cm proximally of the arterial bifurcation at the inguinal fold. We used the highest frequency that reached the far-wall of the artery without compression. Lumen diameter (LD) and IMT were measured in all arteries and intima-media-adventitia thickness (IMAT) was measured in the brachial, radial, and femoral arteries. Adventitia thickness (AT) was calculated as the difference between IMAT and IMT. Measurements were acquired in end-diastole using the leading-edge technique [10]. Each dimension was measured three times using electronic calipers and the mean used in final analyses. We assessed intraobserver and interobserver variability by calculating coefficients of variance (CV) for 10 measurements for each dimension. Intraobserver CV was 0.7–2.5 % for LDs, 4.0–6.0 % for IMTs and 1.0-3.4 % for IMATs. Interobserver CV was 1.6–5.1 % for LDs, 6.0-7.9 % for IMTs and 8.1–15.7 % for IMATs, respectively.

Carotid β-stiffness index (CBSI) [21], carotid distensibility coefficient (CDC) [21], and carotid wall stress (CWS) [22] were calculated using the following formulas:

$$CDC=1000\times \frac{\left(CCLAS-CCLAD\right)}{CCLAD (SBP-DBP)}$$

$$CBSI=\frac{\text{ln}\frac{SBP}{DBP}}{\frac{CCLDS-CCLDD}{CCLDD}}$$

$$CWS\;=\;\frac{MAP\;\times\;CCLDD}{2\;\times\;CCIMT}$$

CCLAS and CCLAD are the carotid artery lumen area in end-systole and end-diastole respectively, CCLDS and CCLDD carotid artery LD in end-systole and end-diastole, respectively and CCIMT the common carotid IMT. SBP and DBP are systolic and diastolic BP and MAP the mean arterial pressure. Carotid-femoral (cfPWV) and carotid-radial pulse-wave velocities (crPWV) were measured at rest in a supine position by a trained technician (Complior Analyse, Alam Medical, Saint-Quentin-Fallavier, France). Mechanosensors were placed at the right carotid, femoral and radial arteries and transit times were recorded twice. Means were used for subsequent calculations. Distances between measurement points were measured using a tape measure to the nearest 0.1 centimeter, and the carotid-femoral distance was multiplied by 0.8. Parallel measurement CVs were 5.7 % for cfPWV and 9.0 % for crPVW. Tolerances for cfPWV and crPWV measurements were 2.0 (IQR: 1.4; 3.3) and 0.4 (IQR: 0.2; 0.6), respectively.

### Blood pressure

BP was measured by a trained technician (Carescape v100, GE Healthcare, Chicago, USA), using appropriately sized cuffs from the right arm with the patient sitting upright, following 1-hour rest during imaging [23]. Means of three consecutive measurements were used in analyses. SBP, DBP, heart rate (HT) and MAP (MAP=(2xDBP + SBP)/3) were recorded and BP Z-scores generated for height [23]. Parallel measurement CVs were 4 % for SBP, 4 % for DBP, and 6 % for HR.

### Anthropometrics and body composition

We examined head, hip and waist circumferences, arm length, midpoint brachial and antebrachial circumferences, leg length, midpoint thigh and calf circumferences using a tape measure. Limb length was measured to the closest 0.5 centimeters, and waist, hip, and other circumferences to the closest 0.1 centimeter. Height and weight were measured to 0.1 centimeter and 0.1 kg, respectively (Seca285, Seca GmBH & Co. KG, Hamburg, Germany).

We calculated the waist hip ratio, body mass index (BMI) and body surface area (BSA) using the Mosteller formula [24] and generated national Z-scores for height, head circumference and BMI in relation to age, and Z-scores for weight in relation to height [18, 19]. LBM and body fat percentage (BF%) was measured with bioelectrical impedance analysis (InBody 7250, Inbody Bldg., Seoul South Korea).

### Lipids, glucose and inflammatory markers

Plasma triglycerides, low- and high-density lipoprotein, total cholesterol and blood glycated hemoglobin were determined from morning venous blood samples following overnight fasting using standard hospital enzymatic assays, fasting plasma glucose using hexokinase assay, serum insulin using immunochemiluminometric assay, and serum high sensitivity C-reactive protein (hs-CRP) using photometric immunochemical assay.

### Questionnaires

We recorded information on diabetes, epilepsy, cerebral palsy and slow intellectual development or intellectual disability, along with parental education, household annual income and parental smoking with standard questionnaires and defined parental smoking as either parent currently smoking regularly or sporadically.

### Data analysis

We report data as mean (SD), median (Q_1_; Q_3_), and N (%) for normally, non-normally distributed and categorical data, respectively. We assessed variable distribution visually and using Shapiro-Wilks test. Far outliers for PWV (median ± 3×IQR) and hs-CRP values outside of laboratory reference values (0.06–3 mg/ml) were excluded.

ANOVA, Kruskal-Wallis or Fisher-Freeman-Halton exact tests were applied to compare groups, as appropriate, following post-hoc tests (Dunnet or Games-Howell, Mann-Whitney U or Z-test for proportions) to check difference between SGA, LGA groups and the AGA control group.

Univariate regressions were performed to assess arterial dimension and stiffness predictors (Supplementary Tables 1–3, Additional files 1–3). Predictors are described in tables and presented grouped into domains: (1) size at birth, (2) sex, (3) age, (4) anthropometrics (5) adiposity, (6) BP, (7) blood lipids (8) blood glucose (9) inflammation and (10) parental smoking. Birth weight, anthropometrics and adiposity were considered main predictors, and the other variables examined exploratorily. The association between arterial dimensions and stiffness and anthropometrics were assessed both for local anthropometrics, such as head and limb circumference, and measurements of body size overall, such as LBM and BSA. For arterial wall layers, the unstandardized coefficient was multiplied by 1000, corresponding to layer thickness in micrometers.

We constructed ANCOVA and multiple regression models to assess the association between birth weight, both as a categorial and a continuous variable, and arterial dimensions, local carotid stiffness and PWV, while adjusting for potential confounders. Based on univariate analyses, sex was identified as a potential confounder and body anthropometrics (BSA and LBM), and adiposity (BF% and BMI) as potential mediators for arterial dimensions and local stiffness and were considered for follow-up in the multiple regression models for arterial dimensions and local stiffness. We chose BSA and LBM as anthropometric predictors for the multiple regression models instead of local anthropometric dimensions, as they reflected body size as a whole, were consistently univariately associated with arterial dimension and local stiffness, and performance in univariate analyses were similar.

For PWV, models with height, LBM, BSA, sex, HR and MAP were explored. Height performed better than BSA or LBM in the univariate analyses and was therefore included in the models. HR and MAP are known confounders for PWV [25]. Multiple regression models were examined for multicollinearity, and a VIF cut-off value of 2.5 chosen for the final models. All tests were two-tailed and a p-value < 0.05 deemed significant.

The initial sample size was determined at the neonate stage based on key cardiovascular dimensions and set to detect a clinically relevant difference of 10–20 % in carotid IMT, with a power of 80 % and an α-error of 5 %. The sample size of the follow-up was determined by the number of patients agreeing to the follow-up.

Data was analyzed using SPSS 27 (IBM, New York, New York, USA) and graphs created with GraphPad Prism 9 (GraphPad Software, San Diego, California, USA).

## Results

### Background, gestation, and growth

**Fig. 2:**
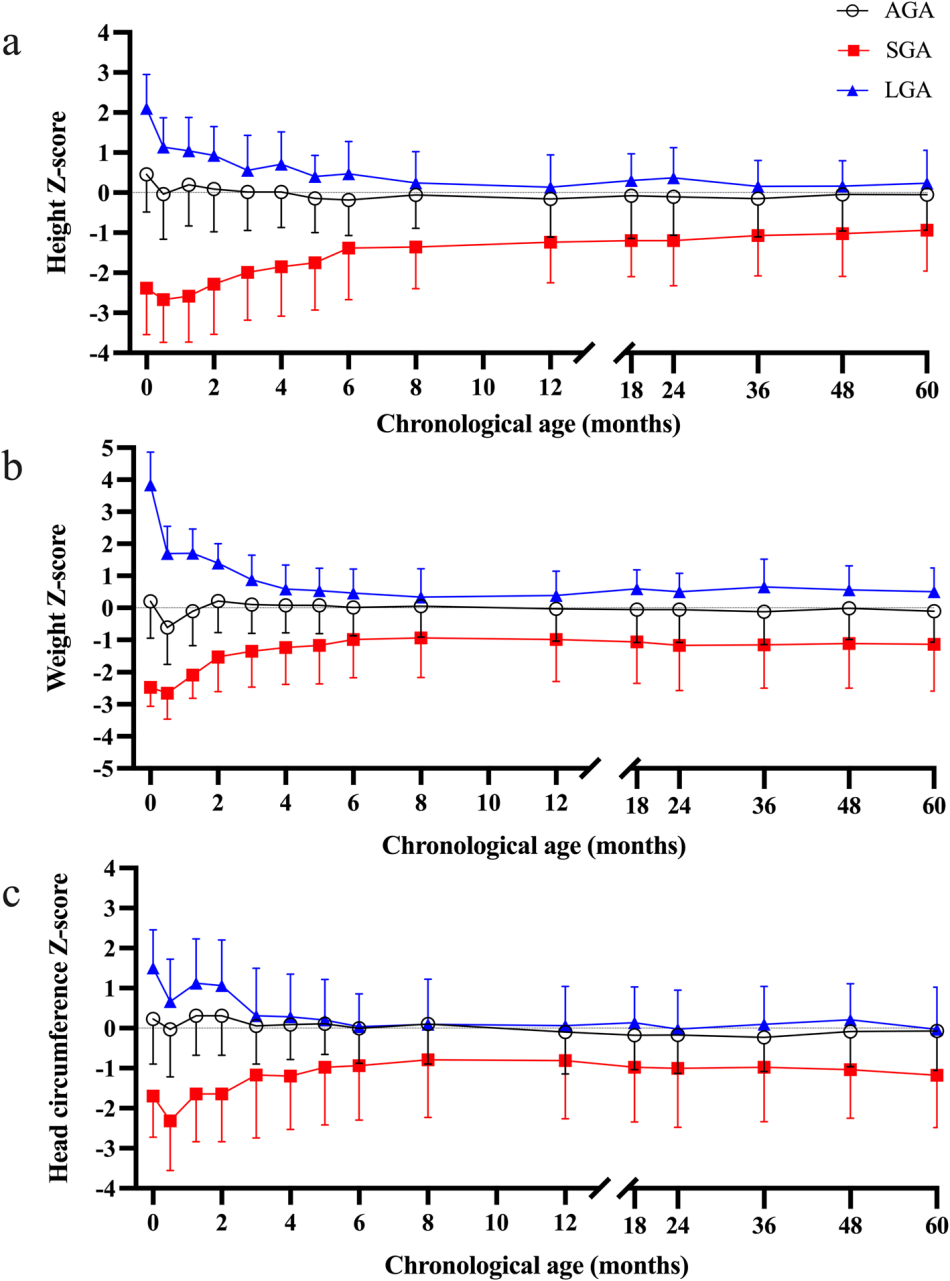
Growth in Z-scores for age from birth to five years of age (mean and SD). Height (**a**), weight (**b**) and head circumference (**c**) have been collected from primary care growth charts. Chronological age, corrected for prematurity, is displayed on the X-axis. AGA indicates appropriate for gestational age, SGA small for gestational age and LGA large for gestational age

Birth and gestational data reflected inclusion criteria with LGA and SGA showing higher numbers of maternal pre-pregnancy diabetes and preeclampsia, respectively. Longitudinal growth for both SGA and LGA groups showed normalization to the population mean for age during the first 6 to 12 postnatal months (Fig. 2). However, SGA remained slightly smaller at 1 y, as measured by weight Z-score for age (Table 1). When examining follow-up participants and non-participants, we found no differences for group composition, sex, gestational parameters or birth weight, height, or head circumference Z-score. In the LGA group, one child had been diagnosed with epilepsy without the need of regular medication, and one with corpus callosum agenesia, while of the AGA children one had been diagnosed with unspecified motor delay and one with mild asymmetric cerebral palsy. None of the children was reported to have a diabetes.

**Table 1:** Background, birth and gestational data, and anthropometric measures and blood work at 5y follow-up

	AGA (N = 48)		SGA (N = 23)		LGA (N = 19)		*p*-value
*Gestation, birth and growth*							
Sex (male/female)	27/21	56 %/44 %	9/14	39 %/61 %	9/10	47 %/53 %	0.376
Age at follow-up (y)	5.8	5.7;5.9	5.8	5.7;6.1	5.8	5.7;5.9	0.776
Maternal pre-pregnancy diabetes	5	5 %	0	0 %	14^***^	74 %	< 0.001
Maternal pre-eclampsia	3	6 %	8^**^	35 %	1	5 %	0.003
Mode of delivery (vaginal/caesarian)^a^	31/16	65 %/33 %	12/11	52 %/48 %	7/12	37 %/63 %	0.087
Gestational age at delivery (weeks)	34.9	34.1;39.3	37.6	34.6;38.1	35.7	34.6;37.6	0.602
Birth height (cm)	47	46; 51	43^***^	42; 46	51^**^	49; 53	< 0.001
Birth height Z-score (cm)	0.43	±0.93	-2.42^***^	±1.19	2.16^***^	0.98	< 0.001
Birth weight (g)	2550	2195;3474	2005^***^	1735:2390	4060^***^	3740;4480	< 0.001
Birth weight (Z-score)	0.06	-0.69;0.85	-2.67^***^	-2.86;-1.97	3.87^***^	3.10;4.13	< 0.001
Birth head circumference (cm)	33.1	± 2.1	31.3^***^	±1.5	35.1^***^	±1.9	< 0.001
Birth head circumference (Z-score)	0.18	0.88	-1.50^***^	±0.86	1.70^***^	±0.75	< 0.001
Weight at 1 y (Z-score)	-0.03	±1.01	-0.98^**^	±1.30	0.39	±0.76	< 0.001
*Anthropometric data at 5y*							
Head circumference (cm)	52.1	±1.4	51.1^**^	±1.4	52.7	±1.3	< 0.001
Head circumference (Z-score)	-0.25	±0.98	-0.94^*^	±1.24	0.29	±0.96	0.001
Height (cm)	116.8	±4.5	111.8^***^	±4.5	117.5	±3.6	< 0.001
Height (Z-score)	-0.03	±0.90	-1.15^***^	±1.06	0.18	±0.65	< 0.001
Weight (kg)	20.1	18.0;22.3	17.9^**^	15.2;20.7	22.6^**^	21.2;23.9	< 0.001
Weight (Z-score)	-0.83	±1.28	-1.48	±2.02	0.38^***^	±1.03	< 0.001
Body surface area (m^2^)	0.81	±0.07	0.75^**^	±0.08	0.86^*^	±0.07	< 0.001
Lean body mass (kg)	17.7	±1.9	15.8^***^	±1.8	18.9^*^	±1.6	< 0.001
Arm length (cm)	36.5	±2.0	35.3^*^	±1.5	36.7	±2.1	0.036
Brachial circumference (cm)	17.8	±1.5	16.9	±1.6	18.9^*^	±1.6	< 0.001
Antebrachial circumference (cm)	17.0	16.4;17.7	16.5^*^	15.1;17.0	17.5	16.8;18.8	0.002
Leg length (cm)	58.9	±3.1	56.4^**^	±3.6	60.1	±3.2	0.001
Thigh circumference (cm)	32.7	±3.2	31.3	±3.0	35.0^**^	±2.6	< 0.001
Calf circumference (cm)	23.0	21.9;24.8	22.0	20.9;23.5	24.8^**^	24.3;25.5	< 0.001
*Adiposity data at 5y*							
Body mass index (kg/m^2^)	14.9	14.0;15.7	13.9	13.1;15.9	16.1^**^	15.3;16.9	< 0.001
Body mass index (Z-score)	-0.80	±1.29	-1.38	±1.92	0.38^***^	±1.04	< 0.001
Body fat (%)	13	9;16	8	4;15	15	12;18	0.046
Waist-hip ratio (no unit)	0.92	0.89;0.95	0.90	0.87;0.94	0.89	0.88;0.93	0.330
*Fasting state blood work at 5y*							
C-reactive protein (mg/l)	0.35	0.23;0.94	0.41	0.19;0.90	0.31	0.21;0.68	0.865
Triglycerides (mmol/l)	0.61	0.47;0.77	0.57	0.51;0.62	0.56	0.50;0.74	0.891
Low-density lipoprotein (mmol/l)	2.48	±0.65	2.38	±0.56	2.69	±0.67	0.370
High-density lipoprotein (mmol/l)	1.44	±0.34	1.50	±0.26	1.55	±0.27	0.501
Total cholesterol (mmol/l)	4.14	±0.73	4.20	±0.60	4.41	±0.71	0.457
Glucose (mmol/l)	5.1	±0.3	5.2	±0.4	4.9	±0.4	0.149
Insulin (mU/l)	5.2	3.3;9.3	3.9	2.6;6.1	5.5	2.2;6.4	0.445
Glycated hemoglobin (mmol/mol)	32	±2	31	±2	33	±2	0.220
*Family smoking and SES at 5y*							
Smoking exposure^b^ (yes/no)	37/11	77 %/23 %	16/5	70 %/22 %	14/4	74 %/21 %	1.000
Annual household income^c^ (≤30 000 €/ 30 001–70 000 €/ >70 000 €)	9/16/23	19 %/33 %/48 %	3/4/14	13 %/18 %/61 %	3/5/9	16 %/26 %/47 %	0.727
Upper secondary school completed^b^ (neither parent/one parent/both parents)	10/21/17	21 %/44 %/35 %	2/8/11	9 %/35 %/48 %	7/4/7	37 %/21 %/37 %	0.179
*AGA* appropriate for gestational age, *LGA* large for gestational age, *SES* socioeconomic status, *SGA* small for gestational age. Data are given as mean ± SD, median Q_1_; Q3 or N and percentages, for normally, non-normally and nominal data, respectively. *p*-values correspond to ANOVA, Kruskal-Wallis or Fisher-Freeman-Halton exact test, as appropriate. Significant differences in post-hoc tests (Dunnet, Games-Howell. Mann-Whitney U or Z-test for proportions) between the SGA or LGA groups and the AGA group are indicated with ^*^, ^**^, ^***^, corresponding to significance levels of < 0.05, < 0.01 and < 0.001 respectively. ^a^Mode of delivery unknown for one AGA patient. ^b^Smoking exposure and parental education status unknown for two SGA patients and one LGA patient. ^c^Annual household income unknown for two SGA and two LGA patients.							

### Anthropometrics, unadjusted arterial dimensions, and arterial health

Anthropometrics varied among groups (Table 1). SGA was consistently smaller than AGA, but with no difference in adiposity. LGA height was similar to AGA, but weight, BMI, BSA, and limb circumferences were higher, along with LBM. BF% was, however, not significantly different between LGA and AGA. Carotid, brachial and femoral LD, and brachial IMT were smaller among SGA, but LGA arterial dimensions were no different from AGA (Table 2). No intimal thickening (> 0.06 mm) was found. We found no differences in blood lipids, glucose, hs-CRP, local carotid stiffness, PWVs or BPs.

**Table 2:** Arterial morphology, function and blood pressure at 5y follow-up

	AGA (N = 48)		SGA (N = 23)		LGA (N = 19)		*p*-value
*Arterial structure*							
Common carotid artery LD (mm)	4.59	±0.28	4.38^**^	±0.27	4.71	±0.27	< 0.001
Common carotid artery IMT (mm)	0.33	±0.04	0.33	±0.03	0.33	±0.04	0.762
Brachial artery LD (mm)	2.36	±0.24	2.12^**^	±0.31	2.44	±0.32	< 0.001
Brachial artery IMT (mm)	0.10	0.09;0.10	0.09^**^	0.08;0.09	0.10	0.09;0.10	0.009
Brachial artery AT (mm)	0.10	±0.02	0.10	±0.01	0.10	±0.02	0.650
Radial artery LD (mm)	1.44	±0.16	1.36^*^	±0.15	1.49	±0.11	0.009
Radial artery IMT (mm)	0.09	0.09;0.10	0.09	0.09;0.10	0.09	0.09;0.10	0.935
Radial artery AT (mm)	0.07	±0.01	0.07	±0.01	0.07	±0.01	0.914
Femoral artery LD (mm)	4.19	3.91;4.46	3.93^***^	3.48;4.07	4.20	3.89;4.60	0.001
Femoral artery IMT (mm)	0.17	0.16;0.19	0.16	0.15;0.18	0.18	0.15;0.21	0.064
Femoral artery AT (mm)	0.17	0.16;0.19	0.16	0.14;0.18	0.18	0.16;0.19	0.081
*Arterial function*							
CBSI (no unit)	2.22	2.02;2.46	2.26	2.10;2.75	2.55	2.11;2.97	0.124
CDC (%/10mm Hg)	12.9	±2.6	12.5	±3.3	11.8	±2.6	0.333
CWS (mmHg)	515	±79	503	±77	523	±85	0.712
Carotid-femoral PWV (m/s)	5.0	4.5;5.4	4.7	4.1;5.2	4.9	4.6;5.2	0.335
Carotid-radial PWV (m/s)	7.4	6.2;8.8	7.2	6.8;8.9	6.8	6.3;8.1	0.451
*Blood pressure*							
Systolic BP (mmHg)	101	±7	102	±10	100	±6	0.790
Diastolic BP (mmHg)	60	57;65	58	56;65	58	55;62	0.780
Systolic BP (Z-score)	0.65	±0.67	0.85	±0.88	0.53	±0.66	0.334
Diastolic BP (Z-score)	0.31	0.00;0.95	0.36	0.15;0.99	0.28	-0.08;0.061	0.538
Mean arterial pressure (mmHg)	74	±5	74	±7	72	±6	0.623
Heart rate (bpm)	84	±12	83	±11	81	±8	0.514

*AGA* appropriate for gestational age, *AT* adventitia thickness, *BP* blood pressure, *CBSI* common carotid beta stiffness index, *CDC* common carotid distensibility coefficient, *CWS* common carotid artery wall stress, *IMT* intima-media thickness, *LD* lumen diameter, *LGA* large for gestational age, *PWV* pulse wave velocity, *SGA* small for gestational age. Data are given as mean ± SD or median and Q1; Q3 for normally and non-normally distributed data, respectively. *p*-values correspond to ANOVA or Kruskal-Wallis test, as appropriate. Significant differences in post-hoc tests (Dunnet and Mann-Whitney U) between the SGA or LGA groups and the AGA group are indicated with ^*^, ^**^, ^***^ corresponding to significance levels of < 0.05, < 0.01, and < 0.001 respectively.

### Predictors of arterial dimensions

**Fig. 3:**
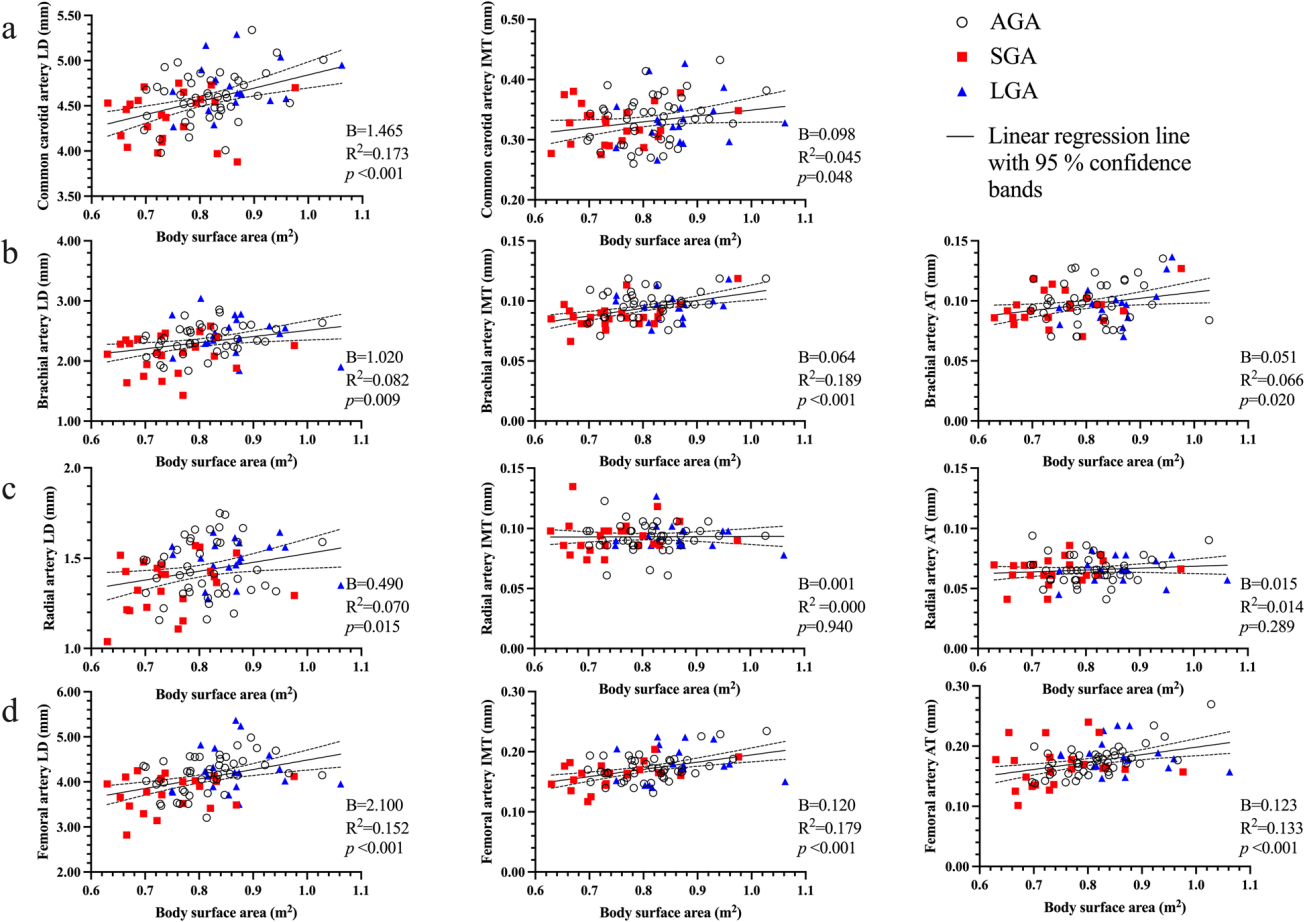
Arterial dimensions plotted against body surface area. Panel (**a**) shows the common carotid, panel (**b**) the brachial, panel (**c**) the radial and panel (**d**) the femoral artery. AGA indicates appropriate for gestational age, SGA small for gestational age, LGA large for gestational age, LD lumen diameter, IMT intima-media thickness, AT adventitia thickness, and B the unstandardized coefficient

The results of the univariate regressions for arterial dimensions are summarized in Supplementary Tables 1–2, Additional files 1 and 2. Birth weight was significantly associated with all LDs and femoral IMT. Male sex was positively associated with carotid and brachial LD and brachial and radial IMT. For LDs, current body size in general, and especially LBM and distal limb circumferences were significant predictors (Fig. 3). LBM consistently performed slightly better than BSA. Distal limb circumferences showed similar associations to LBM, performing slightly better than BSA for all LDs, and slightly better than LBM for carotid and radial LDs (Supplementary Tables 1, Additional file 1). Adiposity, measured as BMI, was weakly associated with carotid and femoral LD only.

Body size was the strongest predictor of arterial wall layers (Fig. 3 and Supplementary Tables 2, Additional file 2), with no consistent difference between LBM and BSA. Distal limb circumferences performed overall similar to LBM and BSA and were consistently more strongly associated with arterial layers compared with proximal limb circumferences. Adiposity was significantly associated with brachial and femoral IMT and AT only. Total cholesterol associated weakly with carotid and femoral IMT and along with low-density lipoprotein with femoral AT. Both glucose and insulin showed weak positive associations with femoral IMT. No relations between arterial dimensions and BP, hs-CRP, or parental smoking were found.

### Predictors of arterial stiffness

Results of univariate analyses for arterial stiffness and function are summarized in Supplementary Tables 3, Additional file 3. cfPWV was associated with body size, most strongly with height. There was a significant association with BF%, but not BMI, as well as SBP, DBP, MAP, hs-CRP and triglycerides. Birth weight, age, male sex or blood glucose did not associate with cfPWV.

No consistent predictors of crPWV, CDC, CBSI or CWS were found.

### Birth weight and arterial dimensions and stiffness, adjusted for body size and sex

We found no differences for arterial dimensions or stiffness between the SGA, LGA and AGA groups, when adjusting for sex and LBM or BSA (Supplementary Tables 4, Additional file 4). The linear associations between birth weight and arterial dimensions were explored with multiple regression models, with birth weight Z-score together with sex, current body size (LBM or BSA), and adiposity (BF% or BMI) entered as predictors. Neither BMI nor BF% remained independent predictors once birth weight Z-score, sex and LBM were adjusted for, and as neither improved model fit measured as adjusted R^2^, they were not included in the final models. Final models for arterial dimensions were created with birth weight, sex and LBM as predictors, as using BSA instead of LBM only improved model fit slightly for two parameters: carotid LD and brachial IMT (adjusted R^2^ 0.287 and 0.233, for both *p* <0.001). This model was significant for carotid, brachial, radial and femoral LD, brachial IMT and femoral IMT and AT (Table 3). Birth weight remained an independent predictor for carotid and brachial LD, while LBM remained a significant predictor for carotid and femoral LD, and male sex for carotid and brachial LDs. Intima-media thicknesses were independently associated only with LBM. For PWV, birth weight, height, MAP and HR were chosen as predictors for the final model (Table 3), as this model had higher adjusted R^2^ than models with BSA or LBM. Sex was not included in the final model, as it neither improved model fit nor showed an independent association. cfPWV was independently associated with height and BP, but not with birth weight. The models were not able to predict local carotid function or crPWV.

**Table 3:** Statistically significant multiple regression models

Dependent	Adjusted R^2^	Model *p*-value	Predictor	β	SE	B	*p*-value
Common carotid artery LD (mm)	0.274	<0.001	Male sex	0.131	0.057	0.222	0.024
			Birth weight, Z-score	0.039	0.013	0.321	0.003
			LBM (kg)	0.032	0.015	0.230	0.038
Brachial artery LD (mm)	0.229	<0.001	Male sex	0.158	0.062	0.262	0.012
			Birth weight, Z-score	0.031	0.014	0.253	0.028
			LBM (kg)	0.030	0.016	0.215	0.069
Radial artery LD (mm)	0.089	0.016	Male sex	-0.033	0.034	-0.106	0.335
			Birth weight, Z-score	0.012	0.008	0.188	0.123
			LBM (kg)	0.016	0.009	0.217	0.085
Femoral artery LD (mm)	0.245	<0.001	Male sex	0.093	0.089	0.103	0.303
			Birth weight, Z-score	0.040	0.020	0.214	0.052
			LBM (kg)	0.075	0.024	0.355	0.002
Brachial artery IMT (mm)	0.210	<0.001	Male sex	0.005	0.002	0.206	0.053
			Birth weight, Z-score	0.000	0.001	-0.056	0.626
			LBM (kg)	0.002	0.001	0.414	<0.001
Femoral artery IMT (mm)	0.207	<0.001	Male sex	0.000	0.005	0.008	0.937
			Birth weight, Z-score	0.000	0.001	-0.001	0.992
			LBM (kg)	0.005	0.001	0.484	<0.001
Femoral artery AT (mm)	0.144	0.001	Male sex	0.003	0.006	0.054	0.608
			Birth weight, Z-score	0.000	0.001	-0.003	0.981
			LBM (kg)	0.005	0.002	0.402	0.001
Carotid-femoral PWV (m/s)	0.224	<0.001	Birth weight, Z-score	0.024	0.031	0.083	0.444
			Height (cm)	0.043	0.016	0.300	0.010
			MAP (mmHg)	0.032	0.014	0.258	0.025
			Heart rate (bpm)	0.011	0.007	0.175	0.112

*AT* = adventitia thickness, *β* = the unstandardized coefficient, *B* = the standardized coefficient, IMT intima-media thickness, *LBM* lean body mass, *LD* lumen diameter, *MAP* mean arterial pressure, *PWV* pulse-wave velocity, *SE* = standard error of β

## Discussion

In this study we assessed the impact of abnormal fetal body growth on arterial morphology, stiffness, and arterial health overall during early childhood. We included postnatal body growth, sex, body composition, and comprehensive measures of cardiovascular risk in the analyses. Although significant independent associations between birth weight and arterial LDs were found, we report no independent associations between restricted or excessive fetal growth and arterial layer thickness or stiffness. In addition, we found no consistent associations between abnormal fetal growth and traditional cardiovascular risks including measures of adiposity, BP, blood lipids, glucose or inflammation. Overall, these findings are consistent with the absence of adverse arterial health effects of fetal programming during early childhood related with abnormal fetal growth.

Increased carotid IMT has, in contrast to our findings, been linked with SGA during early childhood [9]. However, studies are inconclusive, as both increased carotid IMT [26–29] and normal carotid IMT [30], and increased [29] and normal [26] aortic IMT have been reported in SGA children, along with normal carotid IMT in LGA children [31]. We speculate that this could be due to limitations in conventional ultrasound axial resolution applied in earlier studies. The main limiting factor of B-mode ultrasound is axial resolution [32], and conventional ultrasound has been shown to be inadequate for measuring carotid IMT in infants and young children [10, 11]. The arterial wall layer thickness measurement levels reported in study are below the axial resolution of conventional ultrasound for the peripheral arteries and bordering the axial resolution for the carotid artery.

We report no abnormalities in arterial health, including PWV and BP, nor in adiposity, blood glucose or lipids during early childhood following abnormal fetal growth. Increased cfPWV has previously been reported in SGA preschool children [29] and adolescents [33]. Similarly, increased aortic stiffness has been reported in SGA preadolescent [34], but not in young children [27]. Both increased [28] and normal [30] BP have been demonstrated in SGA children. Despite associations between abnormal fetal growth and cardiovascular disease having consistently been reported in adult age, our results suggest that arterial health is unaffected during early childhood or that these changes are so small that they are undetectable even when using improved contemporary very-high resolution methods.

In this study we show that current body size in general, and LBM in particular, is a strong predictor of arterial dimensions in young children, also in the presence of abnormal prenatal growth and postnatal normalization of growth. Our study shows larger arterial dimensions in males compared with females, especially for upper body arterial dimensions. The effect of adiposity on arterial dimensions seems very limited during early childhood, as adding adiposity to the multiple regression analyses did not consistently improve model fit. This is supported by the strong univariate associations with antebrachial, calf and head circumferences, which reflect LBM and are likely less influenced by adiposity during early childhood. This is coherent with our earlier findings in this cohort during the newborn stage [12], as well as in similarly aged children of mothers with obesity and gestational diabetes [14]. However, as there was a significant association between size at birth and carotid and brachial LD in the multiple regression models, this suggest that lumen growth might not follow the overall growth of the body in this setting. Moreover, this disproportion between body and arterial dimensions seemed to be confined to the SGA group as we found no differences between unadjusted LGA and AGA arterial dimensions. However, these results were not observable for arterial wall layer thickness, or radial and femoral artery LD. We realize that the large number of univariate comparisons, combined with a significance level of *p* < 0.05, raises the risk of type I error, especially for the weaker univariate associations. However, as the association between LBM and arterial dimensions were overall highly significant, and this conclusion was supported by the strong associations with distal limb circumferences, the risk of type I error influencing the conclusion is negligible.

Our study is limited by the relatively small sample size and the lack of differentiation between early- and late-onset FGR, as we did not gather information on the time-of-onset of FGR. However, the SGA group filled the criteria for FGR in its entirety, and the normalization of postnatal SGA and LGA body growth trajectories during the first postnatal 6–12 months is consistent with an aberrant fetal growth prenatally. A possible confounder for growth and cardiovascular risk could be prematurity, as prematurity has been associated with similar cardiovascular risk factors as low birth weight [35]. However, as the difference in gestational age between the groups was non-significant, and all three groups contained both premature and full-term children, the effect of prematurity on our conclusions is likely negligible. Bioimpedance derived LBM during early childhood is known to systematically underestimate fat mass [36], limiting the comparisons of LBM assessed with other methods. We also did not assess aortic IMT, which has recently been proposed as marker of early subclinical atherosclerosis in children [37]. However, assessing carotid IMT has long been the standard and is still considered a valuable marker for cardiovascular risk [6, 8, 38]. The strengths of this study include the use of methodology specifically validated for small children, the well characterized longitudinal cohort sample, and the rigid inclusion criteria for abnormal fetal growth with SGA and LGA groups containing the 2.3 most extreme percentiles only.

## Conclusions

Arterial health is not altered during early childhood in the setting of abnormal fetal growth. Arterial dimension growth largely follows postnatal body growth overall and is mainly predicted by current anthropometrics determined by LBM and sex. The present study found no evidence of fetal programming of cardiovascular disease, altered cardiovascular risk or arterial health during early childhood, suggesting that these changes occur later in life.

## Supplementary Information

**Additional file 1: Supplementary table 1.** Showing the results of univariate regressions for arterial lumen diameters.

**Additional file 2: Supplementary table 2.** Showing the results of univariate linear regression results for intima-media thickness and adventitia thickness.

**Additional file 3: Supplementary table 3** The results of univariate linear regression results for arterial stiffness and wall stress.

**Additional file 4: Supplementary table 4.** ANCOVA models comparing study groups adjusting for confounders.

## Data Availability

The datasets used and analyzed during the current study are available from the corresponding author on reasonable request.

## Abbreviations

AGA: appropriate for gestational age
AT: adventitia thickness
BF%: body fat percentage
BMI: body mass index
BP: blood pressure
BSA: body surface area
cfPWV: carotid-femoral pulse wave velocity
crPWV: carotid-radial pulse-wave velocity
CBSI: carotid β-stiffness index
CDC: carotid distensibility coefficient
CV: coefficient of variation
CWS: carotid wall stress
DBP: diastolic blood pressure
hs-CRP: serum high-sensitivity C-reactive protein
FGR: fetal growth restriction
IMT: intima-media thickness
IMAT: intima-media-adventitia thickness
LD: lumen diameter
LBM: lean body mass
LGA: large for gestational age
MAP: mean arterial pressure
PWV: pulse wave velocity
SBP: systolic blood pressure
SGA: small for gestational age
VHRU: very-high resolution ultrasound
