# Supplementary material for: Arterial health during early childhood following abnormal fetal growth
Source: BMC Pediatr. 2022 Jan 14;22:40. doi: 10.1186/s12887-021-02951-2 (PMC8759262; doi:10.1186/s12887-021-02951-2)
Supplement: Supplementary file 1 — Additional file 1: Supplementary table 1. Showing the results of univariate regressions for arterial lumen diameters. [file 12887_2021_2951_MOESM1_ESM.docx]

| **Supplementary table 1.** The results of univariate regressions for arterial lumen diameters**.** | | | | | | | | | | | | | |
| --- | --- | --- | --- | --- | --- | --- | --- | --- | --- | --- | --- | --- | --- |
|  | Domain | Common carotid LD | | | Brachial artery LD | | | Radial artery LD | | | Femoral artery LD | | |
|  |  | B | R^2^ | *p* | B | R^2^ | *p* | B | R^2^ | *p* | B | R^2^ | *p* |
| Birth weight (Z-score) | Size at birth | **0.052** | **0.184** | **<0.001** | **0.042** | **0.123** | **0.001** | **0.018** | **0.083** | **0.008** | **0.073** | **0.151** | **<0.001** |
| Male sex | Sex | **0.169** | **0.082** | **0.007** | **0.189** | **0.100** | **0.004** | -0.016 | 0.003 | 0.642 | 0.157 | 0.030 | 0.107 |
| Age (years) | Age | 0.145 | 0.015 | 0.256 | 0.065 | 0.003 | 0.626 | -0.012 | 0.000 | 0.866 | 0.043 | 0.001 | 0.827 |
| Height (cm) | Anthropometrics | **0.024** | **0.161** | **<0.001** | **0.019** | **0.096** | **0.004** | **0.007** | **0.051** | **0.038** | **0.037** | **0.160** | **<0.001** |
| Body weight (kg) |  | **0.033** | **0.160** | **<0.001** | **0.021** | **0.064** | **0.021** | **0.010** | **0.060** | **0.024** | **0.044** | **0.126** | **<0.001** |
| Body surface area (m^2^) |  | **1.465** | **0.173** | **<0.001** | **1.020** | **0.082** | **0.009** | **0.490** | **0.070** | **0.015** | **2.100** | **0.152** | **<0.001** |
| Lean body mass (kg) |  | **0.059** | **0.184** | **<0.001** | **0.056** | **0.156** | **<0.001** | **0.021** | **0.078** | **0.010** | **0.101** | **0.229** | **<0.001** |
| Head circumference (cm) |  | **0.092** | **0.205** | **<0.001** | - | - | - | - | - | - | - | - | - |
| Brachial circumference (cm) |  | - | - | - | **0.040** | **0.053** | **0.039** | 1.095 | 0.019 | 0.064 | - | - | - |
| Antebrachial circumference (cm) |  | - | - | - | **0.071** | **0.102** | **0.004** | **0.033** | **0.079** | **0.010** | - |  | - |
| Arm length (cm) |  | - | - | - | **0.035** | **0.048** | **0.049** | 0.011 | 0.018 | 0.225 | - | - | - |
| Thigh circumference (cm) |  | - | - | - | - | - | - | - | - | - | **0.036** | **0.065** | **0.018** |
| Calf circumference (cm) |  | - | - | - | - | - | - | - | - | - | **0.085** | **0.162** | **<0.001** |
| Leg length (cm) |  | - | - | - | - | - | - | - | - | - | **0.041** | **0.096** | **0.004** |
| Waist-hip ratio (no unit) | Adiposity | 1.098 | 0.022 | 0.173 | -1.230 | 0.026 | 0.143 | -0.155 | 0.002 | 0.721 | 0.209 | 0.000 | 0.868 |
| Body mass index (kg/m^2^) |  | **0.043** | **0.081** | **0.008** | 0.023 | 0.024 | 0.165 | 0.016 | 0.040 | 0.067 | **0.054** | **0.055** | **0.029** |
| Fat percentage (%) |  | 0.008 | 0.036 | 0.078 | 0.002 | 0.002 | 0.714 | 0.005 | 0.041 | 0.065 | 0.007 | 0.009 | 0.373 |
| SBP (mmHg) | Blood pressure | -0.005 | 0.015 | 0.260 | 0.000 | 0.000 | 0.916 | 0.002 | 0.010 | 0.368 | 0.008 | 0.014 | 0.272 |
| DBP (mmHg) |  | 0.006 | 0.014 | 0.268 | -0.003 | 0.003 | 0.618 | 0.002 | 0.006 | 0.491 | 0.008 | 0.011 | 0.333 |
| SBP (Z-score) |  | **-0.090** | **0.047** | **0.044** | -0.041 | 0.009 | 0.386 | 0.011 | 0.003 | 0.636 | 0.019 | 0.001 | 0.785 |
| DBP (Z-score) |  | 0.021 | 0.002 | 0.692 | -0.055 | 0.013 | 0.306 | 0.005 | 0.000 | 0.844 | 0.025 | 0.001 | 0.754 |
| *B* unstandardized coefficient, *DBP* diastolic blood pressure, *LD* lumen dimension, *SBP* systolic blood pressure. Significant associations (*p <* 0.05) are bolded. | | | | | | | | | | | | | |
