# Supplementary material for: Arterial health during early childhood following abnormal fetal growth
Source: BMC Pediatr. 2022 Jan 14;22:40. doi: 10.1186/s12887-021-02951-2 (PMC8759262; doi:10.1186/s12887-021-02951-2)
Supplement: Supplementary file 2 — Additional file 2: Supplementary table 2. Showing the results of univariate linear regression results for intima-media thickness and adventitia thickness. [file 12887_2021_2951_MOESM2_ESM.docx]

| **Supplementary table 2.** The results of univariate linear regression results for intima-media thickness and adventitia thickness | | | | | | | | | | | | | | | | | | | | | | |
| --- | --- | --- | --- | --- | --- | --- | --- | --- | --- | --- | --- | --- | --- | --- | --- | --- | --- | --- | --- | --- | --- | --- |
|  |  | Common carotid artery IMT | | | Brachial artery IMT | | | Brachial artery AT | | | Radial artery IMT | | | Radial artery AT | | | Femoral artery IMT | | | Femoral artery AT | | |
|  |  | B | R^2^ | *p* | B | R^2^ | *p* | B | R^2^ | *p* | B | R^2^ | *p* | B | R^2^ | *p* | B | R^2^ | *p* | B | R^2^ | *p* |
| Birth weight (Z-score) | Size at birth | 0.427 | 0.001 | 0.805 | 0.688 | 0.021 | 0.199 | 0.372 | 0.003 | 0.609 | -0.107 | 0.000 | 0.844 | 0.011 | 0.000 | 0.983 | **2.340** | **0.056** | **0.028** | 2.245 | 0.037 | 0.076 |
| Male sex | Sex | 0.556 | 0.000 | 0.947 | **7.392** | **0.099** | **0.004** | 2.808 | 0.008 | 0.427 | **6.994** | **0.085** | **0.007** | 4.398 | 0.042 | 0.064 | 5.667 | 0.014 | 0.272 | 7.367 | 0.017 | 0.229 |
| Age (years) | Age | -3.961 | 0.001 | 0.814 | 1.232 | 0.001 | 0.814 | -2.747 | 0.002 | 0.697 | 10.035 | 0.042 | 0.058 | 7.018 | 0.024 | 0.159 | 11.553 | 0.014 | 0.268 | 18.731 | 0.027 | 0.130 |
| Height (cm) | Anthropometrics | **1.675** | **0.045** | **0.049** | **0.861** | **0.123** | **0.001** | 0.624 | 0.036 | 0.089 | 0.068 | 0.001 | 0.808 | **0.558** | **0.059** | **0.027** | **1.825** | **0.140** | **<0.001** | **1.920** | **0.110** | **0.002** |
| Body weight (kg) |  | 2.148 | 0.041 | 0.061 | **1.544** | **0.192** | **<0.001** | **1.262** | **0.071** | **0.016** | -0.010 | 0.000 | 0.978 | 0.242 | 0.007 | 0.469 | **2.657** | **0.164** | **<0.001** | **2.684** | **0.118** | **0.001** |
| Body surface area (m^2^) |  | **98.215** | **0.045** | **0.048** | **64.497** | **0.189** | **<0.001** | **50.986** | **0.066** | **0.020** | 1.200 | 0.000 | 0.940 | 15.450 | 0.014 | 0.289 | **120.236** | **0.179** | **<0.001** | **123.37** | **0.133** | **<0.001** |
| Lean body mass (kg) |  | 3.751 | 0.043 | 0.055 | **2.400** | **0.186** | **<0.001** | 1.340 | 0.032 | 0.110 | 0.449 | 0.006 | 0.467 | **1.147** | **0.049** | **0.046** | **5.320** | **0.230** | **<0.001** | **5.535** | **0.174** | **<0.001** |
| Head circumference (cm) |  | 4.708 | 0.032 | 0.102 | - | - | - | - | - | - | - | - | - | - | - | - | - | - | - | - | - | - |
| Brachial circumference (cm) |  | - | - | - | **2.786** | **0.144** | **<0.001** | **2.275** | **0.075** | **0.014** | -0.556 | 0.006 | 0.482 | -0.007 | 0.000 | 0.992 | - | - | - | - | - | - |
| Antebrachial circumference (cm) |  | - | - | - | **4.171** | **0.201** | **<0.001** | **3.811** | **0.091** | **0.006** | -0.565 | 0.004 | 0.579 | 0.589 | 0.005 | 0.532 | - | - | - | - | - | - |
| Arm length (cm) |  | - | - | - | **1.625** | **0.067** | **0.020** | 0.918 | 0.012 | 0.337 | 0.646 | 0.010 | 0.365 | 0.988 | 0.030 | 0.122 | - | - | - | - | - | - |
| Thigh circumference (cm) |  | - | - | - | - | - | - | - | - | - | - | - | - | - | - | - | **3.408** | **0.214** | **<0.001** | **3.303** | **0.152** | **<0.001** |
| Calf circumference (cm) |  | - | - | - | - | - | - | - | - | - | - | - | - | - | - | - | **4.684** | **0.173** | **<0.001** | **4.952** | **0.148** | **<0.001** |
| Leg length (cm) |  | - | - | - | - | - | - | - | - | - | - | - | - | - | - | - | **3.151** | **0.208** | **<0.001** | **3.022** | **0.144** | **<0.001** |
| Waist-hip ratio (no unit) | Adiposity | -28.375 | 0.002 | 0.789 | 30.022 | 0.010 | 0.379 | 40.148 | 0.010 | 0.381 | 20.716 | 0.005 | 0.541 | 39.332 | 0.021 | 0.194 | -13.355 | 0.000 | 0.840 | -95.899 | 0.018 | 0.221 |
| Body mass index (kg/m^2^) |  | 2.511 | 0.016 | 0.242 | **2.407** | **0.128** | **<0.001** | **2.138** | **0.056** | **0.033** | -0.122 | 0.000 | 0.856 | -0.283 | 0.003 | 0.640 | **3.674** | **0.091** | **0.005** | **3.631** | **0.063** | **0.019** |
| Fat percentage (%) |  | 0.629 | 0.012 | 0.311 | **0.466** | **0.059** | **0.029** | **0.747** | **0.083** | **0.009** | -0.148 | 0.007 | 0.434 | -0.115 | 0.005 | 0.519 | 0.748 | 0.045 | 0.051 | **1.003** | **0.056** | **0.028** |
| SBP, (mmHg) | Blood pressure | 0.510 | 0.009 | 0.374 | 0.223 | 0.019 | 0.212 | 0.096 | 0.002 | 0.689 | 0.170 | 0.011 | 0.344 | -0.080 | 0.003 | 0.645 | 0.607 | 0.033 | 0.092 | **1.001** | **0.064** | **0.018** |
| DBP (mmHg) |  | -0.053 | 0.00 | 0.939 | -0.193 | 0.010 | 0.372 | 0.335 | 0.017 | 0.248 | -0.032 | 0.000 | 0.883 | 0.101 | 0.003 | 0.610 | 0.440 | 0.013 | 0.302 | 0.363 | 0.006 | 0.474 |
| SBP (Z-score) |  | 2.368 | 0.002 | 0.690 | 0.756 | 0.002 | 0.686 | 0.049 | 0.000 | 0.984 | 1.372 | 0.007 | 0.463 | -1.849 | 0.014 | 0.295 | 3.238 | 0.009 | 0.385 | 7.204 | 0.031 | 0.102 |
| DBP (Z-score) |  | -2.895 | 0.002 | 0.672 | -3.088 | 0.026 | 0.147 | 2.428 | 0.009 | 0.398 | -0.195 | 0.000 | 0.929 | 0.377 | 0.000 | 0.848 | 1.643 | 0.002 | 0.698 | 0.666 | 0.000 | 0.895 |
| Triglycerides (mmol/l) | Blood lipids | 8.098 | 0.002 | 0.726 | 1.108 | 0.00 | 0.877 | 8.265 | 0.011 | 0.395 | 6.788 | 0.015 | 0.308 | 3.579 | 0.004 | 0.589 | 11.536 | 0.008 | 0.446 | 9.479 | 0.005 | 0.569 |
| Low-density lipoprotein (mmol/l) |  | 13.447 | 0.047 | 0.064 | -0.511 | 0.001 | 0.821 | 1.719 | 0.005 | 0.577 | 0.610 | 0.001 | 0.775 | 1.828 | 0.011 | 0.387 | 8.313 | 0.044 | 0.078 | **14.905** | **0.117** | **0.003** |
| High-density lipoportein (mmol/l) |  | 17.097 | 0.018 | 0.252 | -2.017 | 0.003 | 0.670 | -0.312 | 0.000 | 0.962 | 0.726 | 0.000 | 0.867 | 2.280 | 0.004 | 0.597 | 14.238 | 0.031 | 0.138 | 17.542 | 0.039 | 0.096 |
| Total cholesterol (mol/l) |  | **13.718** | **0.061** | **0.035** | 0.141 | 0.000 | 0.945 | 2.683 | 0.014 | 0.336 | 0.568 | 0.001 | 0.768 | 1.544 | 0.010 | 0.418 | **8.950** | **0.063** | **0.033** | **12.484** | **0.102** | **0.006** |
| Fasting glucose (mmol/l) | Blood glucose | 2.473 | 0.001 | 0.850 | 3.741 | 0.013 | 0.367 | 7.642 | 0.028 | 0.174 | 2.851 | 0.008 | 0.457 | 4.663 | 0.024 | 0.203 | **17.664** | **0.064** | **0.033** | 0.273 | 0.000 | 0.976 |
| Fasting insulin (mU/l) |  | 0.428 | 0.002 | 0.736 | 0.183 | 0.003 | 0.646 | -0.240 | 0.003 | 0.659 | -0.120 | 0.002 | 0.750 | 0.084 | 0.001 | 0.818 | **2.113** | **0.100** | **0.008** | 1.468 | 0.042 | 0.089 |
| Glycated haemoglobin (mmol/mol) |  | -3.870 | -0.004 | 0.052 | -0.317 | 0.004 | 0.637 | -0.760 | 0.011 | 0.413 | 0.430 | 0.008 | 0.462 | 0.362 | 0.006 | 0.536 | 0.364 | 0.001 | 0.782 | 0.114 | 0.000 | 0.936 |
| C-reactive protein (mg/l) | Inflammation | -2.950 | 0.003 | 0.667 | 2.202 | 0.018 | 0.305 | 4.711 | 0.046 | 0.101 | -0.964 | 0.004 | 0.626 | 0.571 | 0.002 | 0.763 | -1.838 | 0.003 | 0.687 | -0.270 | 0.000 | 0.957 |
| Passive smoking | Smoking | 15.764 | 0.029 | 0.123 | -1.029 | 0.001 | 0.751 | -2.888 | 0.006 | 0.510 | -3.436 | 0.014 | 0.282 | 5.384 | 0.043 | 0.064 | 4.716 | 0.007 | 0.455 | 12.180 | 0.034 | 0.094 |
| *AT* adventitia thickness, *B* unstandardized coefficient, *DBP* diastolic blood pressure, *IMT* intima-media thickness, *SBP* systolic blood pressure. The unstandardized coefficients have been multiplied by 1000, showing the change in micrometers. Significant associations (*p <* 0.05) are bolded. | | | | | | | | | | | | | | | | | | | | | | |
