# Supplementary material for: Arterial health during early childhood following abnormal fetal growth
Source: BMC Pediatr. 2022 Jan 14;22:40. doi: 10.1186/s12887-021-02951-2 (PMC8759262; doi:10.1186/s12887-021-02951-2)
Supplement: Supplementary file 3 — Additional file 3: Supplementary table 3 The results of univariate linear regression results for arterial stiffness and wall stress. [file 12887_2021_2951_MOESM3_ESM.docx]

| **Supplementary table 3.** The results of univariate linear regression results for arterial stiffness and wall stress | | | | | | | | | | | | | | | | | |
| --- | --- | --- | --- | --- | --- | --- | --- | --- | --- | --- | --- | --- | --- | --- | --- | --- | --- |
|  | Domain | CBSI |  |  | CDC |  |  | CWS |  |  | Carotid-radial PWV | | | | Carotid-femoral PWV | | |
|  |  | B | R^2^ | *p* | B | R^2^ | *p* | B | R^2^ | *p* | B | R^2^ | *p* | B | | R^2^ | *p* |
| Birth weight (Z-score) | Size at birth | 0.034 | 0.023 | 0.161 | -0.153 | 0.018 | 0.223 | 4.990 | 0.024 | 0.150 | -0.064 | 0.010 | 0.382 | 0.049 | | 0.028 | 0.151 |
| Male sex | Sex | -0.106 | 0.010 | 0.363 | 0.354 | 0.004 | 0.556 | 13.443 | 0.007 | 0.433 | 0.614 | 0.041 | 0.076 | 0.116 | | 0.007 | 0.471 |
| Age (years) | Age | 0.405 | 0.036 | 0.080 | -1.928 | 0.030 | 0.108 | 42.187 | 0.018 | 0.218 | **1.724** | **0.068** | **0.021** | 0.472 | | 0.025 | 0.174 |
| Height (cm) | Anthropometrics | -0.004 | 0.001 | 0.768 | 0.006 | 0.000 | 0.927 | 1.762 | 0.012 | 0.316 | 0.061 | 0.037 | 0.092 | **0.054** | | **0.142** | **<0.001** |
| Body weight (kg) |  | 0.009 | 0.004 | 0.560 | -0.102 | 0.018 | 0.221 | 2.571 | 0.014 | 0.278 | 0.011 | 0.001 | 0.821 | **0.058** | | **0.096** | **0.006** |
| Body surface area (m^2^) |  | 0.330 | 0.003 | 0.635 | -3.739 | 0.013 | 0.298 | 112.894 | 0.014 | 0.270 | 1.042 | 0.003 | 0.618 | **2.762** | | **0.114** | **0.003** |
| Lean body mass (kg) |  | 0.007 | 0.001 | 0.807 | -0.106 | 0.007 | 0.456 | 3.431 | 0.009 | 0.395 | 0.049 | 0.004 | 0.561 | **0.089** | | **0.073** | **0.018** |
| Head circumference (cm) |  | 0.035 | 0.009 | 0.388 | -0.215 | 0.013 | 0.302 | 5.921 | 0.013 | 0.304 | 0.003 | 0.000 | 0.982 | **0.107** | | **0.057** | **0.038** |
| Brachial circumference (cm) |  | - | - | - | - | - | - | - | - | - | -0.113 | 0.015 | 0.287 | - | | - | - |
| Antebrachial circumference (cm) |  | - | - | - | - | - | - | - | - | - | -0.037 | 0.001 | 0.783 | - | | - | - |
| Arm length (cm) |  | - | - | - | - | - | - | - | - | - | **-** | **-** | **-** | - | | - | - |
| Thigh circumference (cm) |  | - | - | - | - | - | - | - | - | - | - | - | - | 0.038 | | 0.032 | 0.120 |
| Calf circumference (cm) |  | - | - | - | - | - | - | - | - | - | - | - | - | **0.072** | | **0.052** | **0.047** |
| Leg length (cm) |  | - | - | - | - | - | - | - | - | - | - | - | - | **0.064** | | **0.097** | **0.006** |
| Waist-hip ratio (no unit) | Adiposity | -2.301 | 0.029 | 0.115 | 9.238 | 0.017 | 0.223 | 202.928 | 0.010 | 0.349 | 1.764 | 0.002 | 0.691 | 1.241 | | 0.005 | 0.542 |
| Body mass index (kg/m^2^) |  | 0.031 | 0.013 | 0.297 | -0.276 | 0.038 | 0.072 | 4.226 | 0.011 | 0.337 | -0.075 | 0.010 | 0.395 | 0.066 | | 0.037 | 0.097 |
| Fat percentage (%) |  | 0.003 | 0.001 | 0.739 | -0.053 | 0.017 | 0.235 | 1.360 | 0.013 | 0.285 | -0.024 | 0.011 | 0.352 | **0.024** | | **0.058** | **0.037** |
| SBP, office (mmHg) | Blood pressure | - | - | - | - | - | - | - | - | - | -0.023 | 0.011 | 0.370 | **0.028** | | **0.077** | **0.016** |
| DBP, office (mmHg) |  | - | - | - | - | - | - | - | - | - | 0.055 | 0.047 | 0.056 | **0.044** | | **0.149** | **<0.001** |
| SBP, office measurement (Z-score) |  | - | - | - | - | - | - | - | - | - | -0.342 | 0.023 | 0.186 | 0.205 | | 0.040 | 0.084 |
| DBP, office measurement (Z-score) |  | - | - | - | - | - | - | - | - | - | 0.455 | 0.032 | 0.119 | **0.366** | | **0.102** | **0.005** |
| MAP, office (mmHg) |  | - | - | - | - | - | - | - | - | - | 0.031 | 0.013 | 0.327 | **0.048** | | **0.152** | **<0.001** |
| HR, office (bpm) |  | - | - | - | - | - | - | - | - | - | -0.007 | 0.002 | 0.681 | 0.014 | | 0.046 | 0.062 |
| Triglycerides (mmol/l) | Blood lipids | **0.658** | **0.057** | **0.042** | **-3.672** | **0.070** | **0.023** | 59.138 | 0.024 | 0.194 | -0.312 | 0.002 | 0.738 | **0.960** | | **0.064** | **0.041** |
| Low-density lipoprotein (mmol/l) |  | 0.154 | 0.031 | 0.139 | -0.724 | 0.027 | 0.165 | -27.246 | 0.049 | 0.059 | 0.523 | 0.047 | 0.075 | 0.190 | | 0.025 | 0.208 |
| High-density lipoprotein (mmol/l) |  | 0.025 | 0.000 | 0.905 | -0.196 | 0.000 | 0.854 | -22.592 | 0.008 | 0.446 | **1.343** | **0.085** | **0.016** | 0.027 | | 0.000 | 0.925 |
| Total cholesterol (mol/l) |  | 0.103 | 0.017 | 0.269 | -0.663 | 0.028 | 0.156 | -19.167 | 0.030 | 0.140 | **0.599** | **0.077** | **0.022** | 0.171 | | 0.025 | 0.204 |
| Fasting glucose (mmol/l) | Blood glucose | -0.080 | 0.003 | 0.662 | -0.376 | 0.002 | 0.686 | 29.991 | 0.019 | 0.247 | 0.642 | 0.027 | 0.182 | 0.305 | | 0.024 | 0.219 |
| Fasting insulin (mU/l) |  | -0.015 | 0.010 | 0.404 | -0.007 | 0.000 | 0.936 | 2.883 | 0.019 | 0.255 | 0.051 | 0.017 | 0.294 | 0.040 | | 0.041 | 0.106 |
| Glycated hemoglobin (mmol/mol) |  | 0.008 | 0.001 | 0.780 | -0.144 | 0.015 | 0.310 | **8.977** | **0.070** | **0.025** | -0.002 | 0.000 | 0.977 | 0.033 | | 0.011 | 0.405 |
| C-reactive protein (mg/l) | Inflammation | 0.101 | 0.016 | 0.325 | -0.671 | 0.028 | 0.190 | 10.222 | 0.010 | 0.442 | 0.086 | 0.002 | 0.742 | **0.281** | | **0.084** | **0.029** |
| Passive smoking | Smoking | 0.012 | 0.000 | 0.936 | 0.144 | 0.000 | 0.846 | -26.142 | 0.020 | 0.198 | 0.615 | 0.029 | 0.141 | -0.166 | | 0.010 | 0.398 |
| *B* unstandardized coefficient, *CBSI* common carotid beta stiffness index, *CDC* common carotid distensibility coefficient, *CWS* carotid-wall stress, *DBP* diastolic blood pressure, *HR* heart rate, *MAP* mean arterial pressure; *PWV* pulse wave velocity, *SBP* systolic blood pressure. Significant associations (*p <* 0.05) are bolded. | | | | | | | | | | | | | | | | | |
