# Supplementary material for: Arterial health during early childhood following abnormal fetal growth
Source: BMC Pediatr. 2022 Jan 14;22:40. doi: 10.1186/s12887-021-02951-2 (PMC8759262; doi:10.1186/s12887-021-02951-2)
Supplement: Supplementary file 4 — Additional file 4: Supplementary table 4. ANCOVA models comparing study groups adjusting for confounders. [file 12887_2021_2951_MOESM4_ESM.docx]

| **Supplementary table 4.** ANCOVA models comparing study groups adjusting for confounders, with models for LD and IMT and statistically significant models for AT and arterial stiffness shown. | | | | | | | |
| --- | --- | --- | --- | --- | --- | --- | --- |
| Variable | Adjusted R^2^ | Model *p*-value | Predictor | Estimated marginal means (95% CI) | | | *p*-value |
| Common carotid artery LD (mm) | 0.247 | <0.001 | Group | AGA | 4.57 | (4.50-4.65) | 0.079 |
|  |  |  |  | SGA | 4.46 | (4.34-4.57) |  |
|  |  |  |  | LGA | 4.67 | (4.54-4.79) |  |
|  |  |  | Male sex | | | | 0.038 |
|  |  |  | Lean body mass (kg) | | | | 0.033 |
| Brachial artery LD (mm) | 0.230 | <0.001 | Group | AGA | 2.34 | (2.26-2.42) | 0.052 |
|  |  |  |  | SGA | 2.18 | (2.06-2.30) |  |
|  |  |  |  | LGA | 2.41 | (2.28-2.54) |  |
|  |  |  | Male sex | | | | 0.019 |
|  |  |  | Lean body mass (kg) | | | | 0.112 |
| Radial artery LD (mm) | 0.092 | 0.020 | Group | AGA | 1.44 | (1.40-1.49) | 0.145 |
|  |  |  |  | SGA | 1.37 | (1.30-1.44) |  |
|  |  |  |  | LGA | 1.48 | (1.40-1.55) |  |
|  |  |  | Male sex | | | | 0.278 |
|  |  |  | Lean body mass (kg) | | | | 0.173 |
| Femoral artery LD (mm) | 0.239 | <0.001 | Group | AGA | 4.15 | (4.03-4.27) | 0.117 |
|  |  |  |  | SGA | 3.92 | (3.73-4.10) |  |
|  |  |  |  | LGA | 4.15 | (3.96-4.34) |  |
|  |  |  | Male sex | | | | 0.399 |
|  |  |  | Lean body mass (kg) | | | | 0.003 |
| Common carotid artery IMT (mm) | 0.003 | 0.379 | Group | AGA | 0.33 | (0.32-0.34) | 0.820 |
|  |  |  |  | SGA | 0.33 | (0.32-0.35) |  |
|  |  |  |  | LGA | 0.33 | (0.31-0.34) |  |
|  |  |  | Male sex | | | | 0.629 |
|  |  |  | Lean body mass (kg) | | | | 0.058 |
| Brachial artery IMT (mm) | 0.221 | <0.001 | Group | AGA | 0.10 | (0.09-0.10) | 0.289 |
|  |  |  |  | SGA | 0.09 | (0.09-0.10) |  |
|  |  |  |  | LGA | 0.09 | (0.09-0.10) |  |
|  |  |  | Male sex | | | | 0.042 |
|  |  |  | Lean body mass (kg) | | | | 0.005 |
| Radial artery IMT (mm) | 0.055 | 0.077 | Group | AGA | 0.09 | (0.09-0.09) | 0.345 |
|  |  |  |  | SGA | 0.10 | (0.09-0.10) |  |
|  |  |  |  | LGA | 0.09 | (0.09-0.10) |  |
|  |  |  | Male sex | | | | 0.011 |
|  |  |  | Lean body mass (kg) | | | | 0.711 |
| Femoral artery IMT (mm) | 0.195 | <0.001 | Group | AGA | 0.17 | (0.17-0.18) | 0.896 |
|  |  |  |  | SGA | 0.17 | (0.16-0.18) |  |
|  |  |  |  | LGA | 0.17 | (0.16-0.19) |  |
|  |  |  | Male sex | | | | 0.803 |
|  |  |  | Lean body mass (kg) | | | | <0.001 |
| Femoral artery AT (mm) | 0.136 | 0.003 | Group | AGA | 0.17 | (0.17-0.18) | 0.929 |
|  |  |  |  | SGA | 0.18 | (0.16-0.19) |  |
|  |  |  |  | LGA | 0.18 | (0.16-0.19) |  |
|  |  |  | Male sex | | | | 0.687 |
|  |  |  | Lean body mass (kg) | | | | <0.001 |
| Carotid-femoral PWV (m/s) | 0.210 | <0.001 | Group | AGA | 4.9 | (4.7-5.1) | 0.862 |
|  |  |  |  | SGA | 4.8 | (4.5-5.1) |  |
|  |  |  |  | LGA | 5.0 | (4.6-5.3) |  |
|  |  |  | Height (cm) | | | | 0.015 |
|  |  |  | Heart rate, office (bpm) | | | | 0.116 |
|  |  |  | Mean arterial pressure, office (mmHg) | | | | 0.024 |
| *AGA* appropriate for gestational age, *AT* adventitia thickness, *CI* confidence interval, *IMT*, intima-media thickness, *LD* lumen dimension, *LGA* large for gestational age, *PWV* pulse wave velocity, *SGA* small for gestational age. | | | | | | | |
